# The Green Synthesis of MgO Nano-Flowers Using* Rosmarinus officinalis* L. (Rosemary) and the Antibacterial Activities against* Xanthomonas oryzae* pv.* oryzae*

**DOI:** 10.1155/2019/5620989

**Published:** 2019-02-17

**Authors:** Yasmine Abdallah, Solabomi Olaitan Ogunyemi, Amro Abdelazez, Muchen Zhang, Xianxian Hong, Ezzeldin Ibrahim, Afsana Hossain, Hatem Fouad, Bin Li, Jianping Chen

**Affiliations:** ^1^State Key Laboratory of Rice Biology, Ministry of Agriculture Key Lab of Molecular Biology of Crop Pathogens and Insects, Institute of Biotechnology, Zhejiang University, Hangzhou 310058, China; ^2^Department of Plant Pathology, Faculty of Agriculture, Minia University, Minya 61519, Egypt; ^3^Department of Dairy Microbiology, Animal Production Research Institute, Agriculture Research Centre, Dokki, Giza 12618, Egypt; ^4^Department of Plant Pathology, Plant Pathology, Research Institute, Agricultural Research Centre, Cairo 12619, Egypt; ^5^Department of Field Crop Pests, Plant Protection Research Institute, Agricultural Research Centre, Cairo 12619, Egypt; ^6^Institute of Plant Virology, Ningbo University, Ningbo 315211, China

## Abstract

Recently, the use of herbs in the agriculture and food industry has increased significantly. In particular,* Rosmarinus officinalis* L. extracts have been reported to have strong antibacterial properties, which depend on their chemical composition. The present study displayed a biological method for synthesis of magnesium oxide (MgO) nano-flowers. The nano-flowers are developed without using any catalyst agent. Aqueous Rosemary extract was used to synthesize MgO nano-flowers (MgONFs) in stirring conditions and temperature at 70°C for 4 h. The mixture solution was checked by UV-Vis spectrum to confirm the presence of nanoparticles. The MgO nano-flowers powder was further characterized in this study by the X-ray diffraction, scanning electron microscopy, transmission electron microscopy, and Fourier transform infrared spectroscopy. In addition, bacteriological tests indicated that MgO nano-flowers significantly inhibited bacterial growth, biofilm formation, and motility of* Xanthomonas oryzae* pv.* oryzae*, which is the causal agent of bacterial blight disease in rice. The electronic microscopic observation showed that bacterial cell death may be mainly due to destroy of cell integrity, resulting in leakage of intracellular content. As recommended, the use of Rosemary extract is an effective and green way to produce the MgO nano-flowers, which can be widely used in agricultural fields to suppress bacterial infection.

## 1. Introduction

*Xanthomonas oryzae* pv.* oryzae* (Xoo), a Gram-negative rod bacteria, is the causal agent of leaf blight disease, which has been reported in most of rice producing countries. The production of rice is adversely affected by this bacterial disease, resulting in annual productivity losses of 10-50% and even 100% under severe conditions [1, 2]. Notably, the nanoparticles may be able to play a pivotal role in rice production due to their antibacterial agents, small size, structure, and surface characteristics [3].

Recently, the green synthesis of nanoparticles has gained considerable attention. In this regard, plant extracts and natural resources such as microorganisms and enzymes have been found to be worthy alternative reagents in the synthesis of nanoparticles [4, 5]. The use of alternative green materials has many advantages such as low energy consumption and moderate operating conditions (e.g., pressure and high temperature) without the use of any toxic chemicals or catalysts [6]. Thus, it could be inferred that the synthesis of nanoparticles using plant extracts is promising because of their simplicity, cost-effectiveness, and environmental aspects [7, 8].

On the contrary, the chemical and physical methods are quite expensive and probably hazardous to the environment due to the use of toxic and unsafe chemicals. Therefore, green synthesis techniques have been developed by using many organisms such as yeast, mold, algae, bacteria, sugars, and polymers (chitosan), or plant extracts for the synthesis of nanoparticles. The reducing agents for plant extracts include various water-soluble plant metabolites (e.g., alkaloids, phenolic compounds, and terpenoids) and co-enzymes [8].

The inorganic materials such as metals and metal oxides have attracted a lot of attention over the past decade due to their ability to withstand synthesis conditions [9–11]. Several metal oxides such as TiO_2_, NiO, ZnO, MgO, and CuO have been used in a wide range of different applications, not only because they are stable under operating conditions but also because of their safety [12].

Interestingly, the MgO nanoparticles (MgONPs) have been mentioned in various applications [13–18]. In particular, MgONPs have demonstrated a great ability to distort cell membranes of* E. coli*, which lead to cell death due to leakage of intracellular content [19, 20]. However, little information was available about the antimicrobial properties of MgONPs against phytopathogenic bacteria. It is noteworthy that MgO nano-flowers (MgONFs) have great potential as an antagonist against Xoo, the pathogen of rice bacterial blight. The main objective of the present study was to synthesize and characterize the MgONFs with strong antibacterial activity against the bacterial blight pathogen of rice.

## 2. Materials and Methods

### 2.1. Preparation of Rosmarinus officinalis Extract

Fresh flowers of Rosemary were obtained from the local market. Rosemary must be fully dried and then placed in a domestic mixer (Media, China) for 10 s. Particle size distribution was determined on a vibrating sieve shaker (Media, China) using screen sieves for the standard Tyler-16 + 80 series. The particles were used for mesh -20 and +32. The ground particles were stored under vacuum and maintained in a domestic freezer (Haier, China) below –10°C. One gram of Rosemary powder was boiled with 100 ml of distilled water at 70°C for 4 h. The extract was filtered through the Whatman filter paper No. 1 and, finally, the yellow-brown extract was collected for further experiments.

### 2.2. Green Synthesis of MgONFs

**Figure 1:**
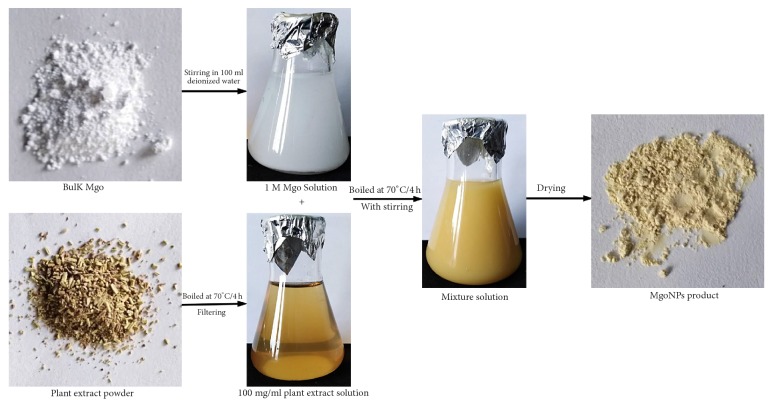
Schematic of green synthesis of MgONFs using* Rosmarinus officinalis* L. (Rosemary).

The synthesis of MgONFs was carried out according to the method of Srivastava et al. [21]. In brief, 100 ml of Rosemary extract was mixed with 100 ml aqueous solution of 1.0 mM MgO solution and stirred continuously at 600 rpm at 70°C for 4 h using a magnetic stirrer (HJ-3 Thermostatic Magnetic Stirrer, Jiangsu, China). Following separation by centrifugation at 5000 rpm for 15 min, the precipitate was washed with distilled water and dried in ALPHA 1-2/LD-Plus vacum. The method was concluded as seen in diagram (Figure 1).

### 2.3. Characterization of MgONFs

The presence of nanoparticles in the solution mixture was verified using a UV-VIS spectrometer according to the method of Nemade et al. [11]. Furthermore, characterization of the MgONFs were carried out as described by Li et al. [22]. In detail, the purity of the phase was determined by X-ray diffraction (XRD) analysis using an XPert PRO diffractometer (Holland) with a detector operating under a voltage of 45.0 kV and a current of 20.0 mA with Cu-K*α* radiation. The recording range of 2*θ* was 20° to 80°. The average crystallite size from XRD was calculated using Scherrer equation [23]. Fourier-transform infrared (FTIR) spectra were recorded in the range of 4000-400 cm^−1^ on a Vector 22 spectrometer (Bruker, Germany). The morphology was examined with a TM-1000 scanning electron microscope (SEM) (Hitachi, Japan) and a JEM-1230 transmission electron microscope (TEM) (JEOL, Japan).

### 2.4. Bacterial Strains and Growth Conditions

Xoo strain GZ 0005 was obtained from Key Laboratory of Rice Biology and Ministry of Agriculture Key Lab of Molecular Biology of Crop Pathogens and Insects, Institute of Biotechnology, Zhejiang University, Hangzhou, China. The overnight cultured strain was inoculated in liquid or solid (1.5% w/v agar) nutrient broth (NB) and agar (NA) and then incubated at 30°C in a shaking incubator at 180 rpm.

### 2.5. Antibacterial Activity of MgONFs against Xoo

The antibacterial activities of MgO and MgONFs at concentrations of 4, 8, and 16 *μ*g/ml against Xoo strain GZ 0005 were assessed by measuring clear zones formed around bacterial colonies as described by Jarrar et al. [24] with some modifications. In brief, 50 *μ*l of the MgO and MgONFs solutions were placed into agar wells of 6-mm in diameter in NA medium, which was seeded with bacteria to give a final concentration of approximately 10^8^ CFU/ml. The plates were kept at 4°C for 3 h to permit diffusion of the assay materials. After incubation at 30°C for 28 h, the diameters of clear zones around the agar well were measured.

Furthermore, the antibacterial activity of MgONFs against Xoo strain GZ 0005 was determined in 96-well microtiter plates by measuring the OD_600_ using a microplate reader, which was carried out as described by Consoli et al. [25]. In brief, each well of 96-well microtiter plates was filled with 100 *μ*l solution of MgONFs, which were suspended in NB broth to give a final concentration of 4, 8, and 16 *μ*g/ml. Then, 1 *μ*l suspension of bacteria in the logarithmic growth stage (OD_600_ = 1.0) was added into solution of MgONFs. Bacterial initial concentration (OD600) was determined using a spectrophotometer (Thermo Scientific Fisher Company, Waltham, MA, USA). The test plates were incubated for 48 h at 30°C without shaking. All experiments were performed in triplicate, while NB was used as the negative control. The inhibitory effect was assayed by comparing the OD_600_, which was carried out as described by Shi et al. [26].

### 2.6. Effect of MgONFs on Biofilm Formation of Xoo

Biofilm formation of Xoo strain GZ 0005 was determined by measuring the OD_570_ value using a Microplate photometer (Thermo Fisher Scientific Inc., Waltham, MA, USA) according to the method of Singh et al. [27] with some modifications. In brief, 1.0 *μ*l of bacterial cultures (OD_600_ = 1.0) in the mid-exponential growth phase was added to 199 *μ*l solution of MgONFs, which were dissolved into NB to obtain a final concentration of 0, 4, 8, and 16 *μ*g/ml. After incubating the mixture suspension at 30°C for 48 h without shaking, each well was added with 0.1% crystal violet (Sigma, USA), while the unbound crystal violet was discarded. Ethanol (95%) was used to absorb crystal violet from the biofilm. All experiments were carried out in triplicate.

### 2.7. Effect of MgONFs on Swimming Motility of Xoo

Effect of MgONFs on the swimming motility of Xoo strain GZ 0005 was determined by incubating bacteria on 0.3% (w/v) NA medium, which was carried out according to the method of Lai et al. [28] with some modifications. In brief, MgONFs were added into 0.3% (w/v) NA medium to give a final concentration of 4, 8, and 16 *μ*g/ml. Then, the overnight cultured bacteria were spotted on the centre of the medium plates. After incubating the plates at 30°C for 2 d, the diameter of motility was measured. All experiments were carried out in triplicate.

### 2.8. Effect of MgONFs on Cellular Integrity of Xoo

Effect of MgONFs on cellular integrity of Xoo strain GZ 0005 was determined according to the method of Li et al. [29] with some modifications. In brief, the overnight cultured bacteria were inoculated into MgONFs solution of 2 *μ*g/ml to give a final bacterial concentration of 10^8^ CFU/ml and then the mixture was incubated on a rotary shaker (160 rpm) at 30°C for 2 h. After centrifugation at 8000 g for 10 min at 4°C, bacterial cells were washed twice with 0.1 mol/l phosphate buffered saline (PBS) solution at pH 7.2 and fixed with 2.5% (v/v) glutaraldehyde. Post-fixation was carried out in 1% (w/v) osmium tetroxide in 0.1 mol/l PBS for 1 h at room temperature followed by dehydration at 4°C for 15 min in a graded series of ethanol solutions and embedded in Epon812, a low-viscosity embedding medium. Thin sections of the specimens were cut with a diamond knife on an Ultra microtome (Super Nova; Reichert-Jung Optische Werke, Vienna, Austria) and the sections were double-stained with saturated uranyl acetate and lead citrate. The grids were examined with an H-7000FA transmission electron microscope (Hitachi, Tokyo, Japan) at an operating voltage of 75 kV.

### 2.9. Statistical Analysis

Statistical analysis was performed using SAS system software (version 9.1, SAS Institute, Cary, NC, USA). All the data in each experiment was subjected to one-way analysis of variance (ANOVA) and means were compared by Duncan's multiple range test. Values of* P* < 0.05 were considered statistically significant.

## 3. Results

### 3.1. Characterization of MgONFs by UV-VIS Spectroscopy

**Figure 2:**
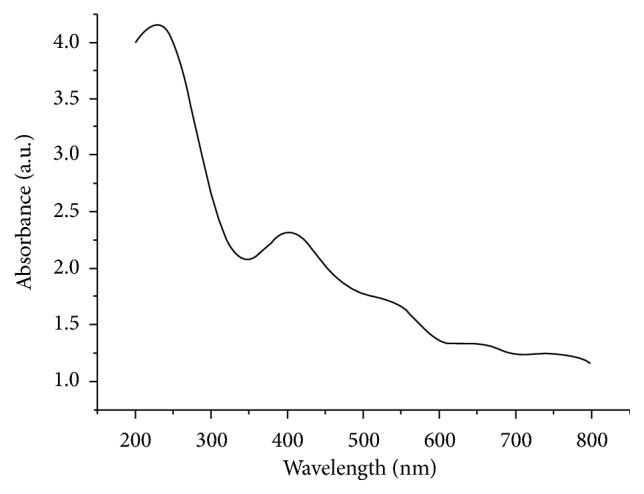
UV-Vis spectrum of MgONFs.

In order to make sure that the MgO nanoparticles were successfully synthesized, the UV-VIS technique was used to determine the structural characterization of the synthesized MgONFs. As shown in Figure 2, the absorbances at wavelengths ranging from 200 nm to 800 nm were determined in this study based on the result of UV-VIS spectroscopy. It was found that there are two peaks of UV-VIS spectrum in the range of 200-800 nm. The highest peak was observed at 250 nm, which can be attributed to the MgO nanoparticles.

### 3.2. Characterization of MgONFs by XRD Analysis

**Figure 3:**
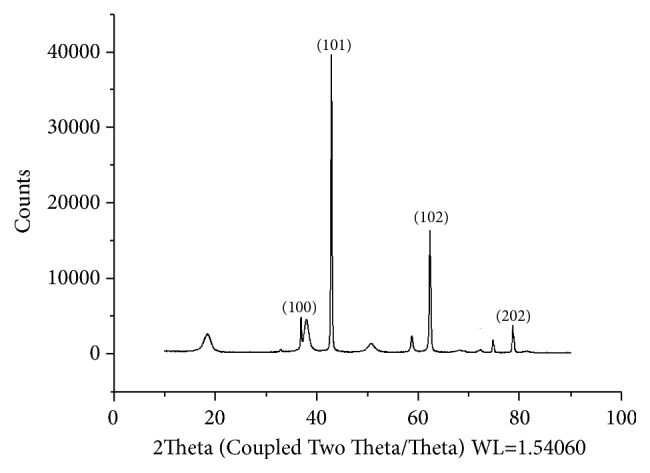
XRD patterns of MgONFs.

MgONPs were obtained as powder and the result of the XRD showed a little amount of impurities (Figure 3). The structure of the MgONPs looks similar to that of flowers. The XRD pattern of the MgONPs synthesized in this study displayed four sharp diffraction peaks at 38.048 (100), 41.095 (101), 78.628 (202), and 62.342 (102). In particular, the latter three peaks have been reported to be indexed to a hexagonal crystal structure of MgO [30, 31]. Furthermore, Safaei-Ghomia et al. [32] illustrated that Debye Scherer's formula was able to be used to find the particle size of synthesized MgONPs (0.9*λ*/(B*∗*cos⁡*θ*) and the size was found to be 25 and 27 nm. In contrast, the size of MgONPs synthesized in this study was found to be at an average of 8.8 nm.

### 3.3. Characterization of MgONFs by FTIR Analysis

**Figure 4:**
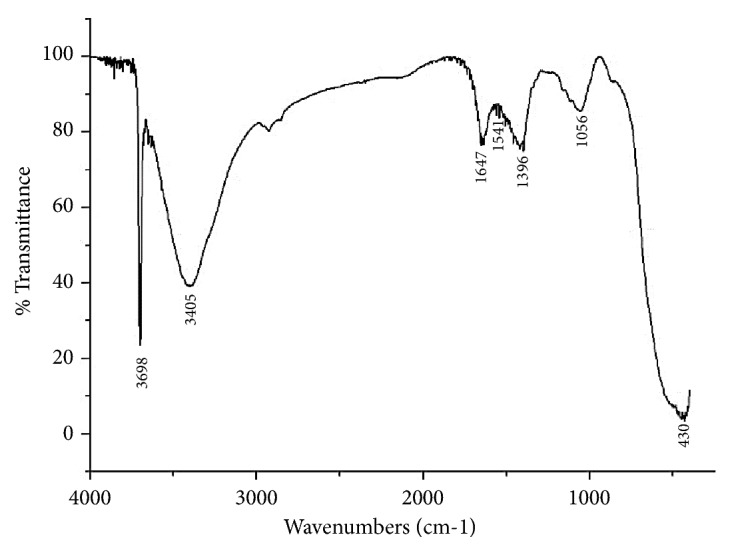
FTIR spectrum of MgONFs.

The FTIR spectrum of MgONFs was shown in Figure 4. There were 6 main bands in the FTIR profile of the MgO nanoflowers synthesized in this study. Based on previous reports [21, 33, 34], these characteristic bands of FTIR can be assigned to various biologically active functional groups. For example, the band at 3405 cm^−1^ is ascribed to the OH stretching. The band at 1647 cm^−1^ is a result of amide group (C=O)NH. The band at 1396 cm^−1^ is the bending vibration of OH bonds. In particular, the band at 430 is due to the stretching vibration of MgO. The band at 3698 cm^−1^ is due to antisymmetric stretching vibration in the Mg(OH)_2_ crystal structure. Therefore, it can be inferred that FTIR analysis can be used to reveal the vibrational frequency of expansion and bending modes of the MgONFs molecules.

### 3.4. Characterization of MgONFs by TEM and SEM

**Figure 5:**
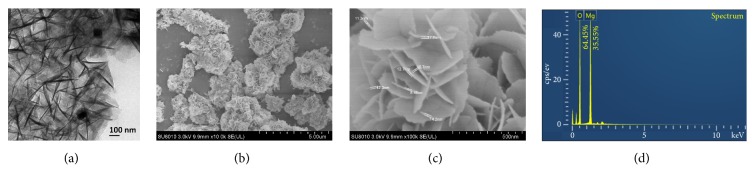
TEM (a), SEM (b and c), observation and EDS spectrum (d) of MgONFs.

Figure 5(a) displayed the shape and size of MgONFs synthesized in this study based on the TEM observation, while SEM observation provided further insight into the shape and size of the synthesized nanoparticles. It has been also found that the MgONFs particles were well dispersed, which was illustrated by the result of the SEM observation. Representative SEM micrographs of the synthesized nanoparticles were shown in Figures 5(b) and 5(c). In general, the SEM images exhibited that the morphological surface were in the form of assemblies of nanoparticles having round shape. Meanwhile, the MgONFs were uniformly distributed over the entire surface. The size of nanoparticles as shown in SEM images was less than 20 nm. The presence of Mg and O elements in the synthesized nanoparticles can be confirmed by EDS, where the result of EDS indicated that the average percentages of Mg and O are 35.55 and 64.45, respectively (Figure 5(d)).

### 3.5. Antibacterial Activity and Mechanism of MgONFs

**Figure 6:**
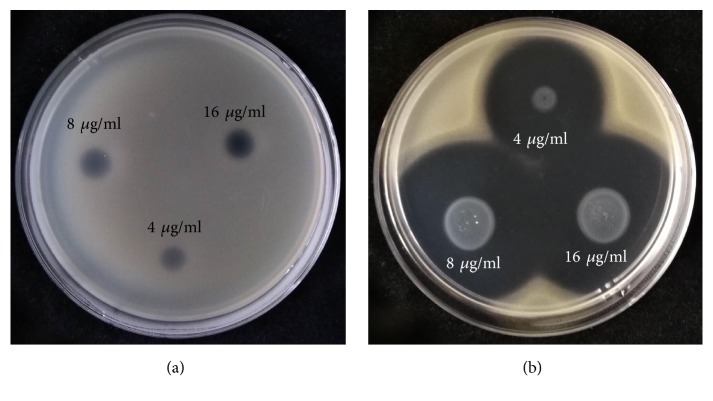
Antibacterial activity of MgO (a) and MgONFs (b) against Xoo strain GZ 0005.

Result from this study indicated that MgONFs at three different concentrations had strong antibacterial activity against Xoo strain GZ 0005 on NA plates. Indeed, the diameter of the inhibition zone was 3.2, 4.4, and 5.1 cm, respectively, in the presnece of MgONFs at the concentrations of 4, 8, and 16 *μ*g/ml (Figure 6). In contrast, the diameter of the inhibition zone was 0.7 cm in the presnece of MgO (Figure 6). Thus, it can be inferred that the MgONFs synthesized in this study had stronger antibacterial activity than that of MgO.

**Figure 7:**
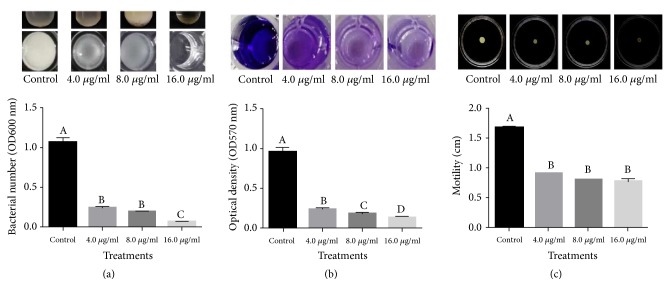
Effects of MgONFs on growth (a), biofilm formation (b), and motility (c) of Xoo strain GZ 0005.

Furthermore, data obtained from Figure 7 indicated that MgONFs at three different concentrations had strong inhibitory effect on the growth, biofilm formation, and swimming of Xoo strain GZ 0005 after incubation of 48 h. Indeed, there were a 77.1, 87.1, and 92.2%, respectively, reduction in the value of OD_600_ in the presnece of MgONFs at the concentrations of 4, 8, and 16 *μ*g/ml (Figure 7(a)). This result also showed that MgONFs at the concentrations of 4, 8, and 16 *μ*g/ml caused a reduction of 77.7, 81.5, and 86.7%, respectively, in the value of OD_570_ compared to the control (Figure 7(b)), while MgONFs at the concentrations of 4, 8, and 16 *μ*g/ml showed a inhibition rate of 44.8, 52.9, and 58.8%, respectivley, in bacterial motility compared to the control (Figure 7(c)).

**Figure 8:**
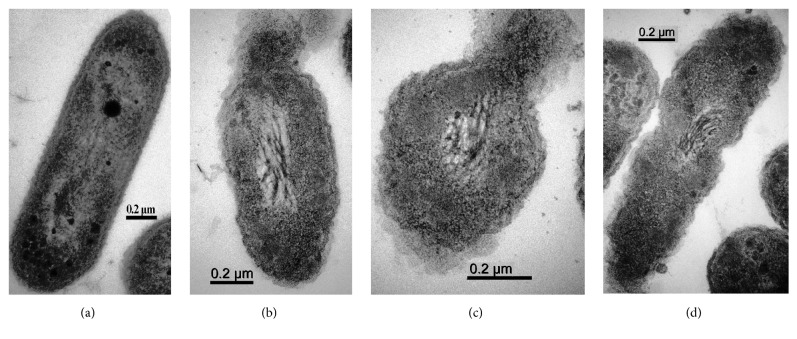
TEM graphs of Xoo strain GZ 0005 cells (a) and treated with 4 *μ*g/ml MgONFs (b–d).

In addition, Figure 8 presented the TEM images of Xoo strain GZ 0005 treated with MgONFs solution at the concentrations of 4 *μ*g/ml. In general, it could be obviously observed that bacterial cells treated with distilled water appeared to be intact. In contrast, destruction of cell wall was observed in bacterial cells treated with the MgONFs (Figure 8). This result is consistent with the data of the antibacterial activty of MgONFs solution. Therefore, it could be suggested that the antibacterial activty of MgONFs against Xoo strain GZ 0005 may be, at least partially, attributed to the destruction of cell wall.

## 4. Discussion

In this study, we successfully synthesized the MgONFs by mixing MgO with the Rosemary extract, which provide a basis for further study on its physical and biochemical characterization. Furthermore, the green synthesis of MgONFs has been confirmed by the characteristic absorption peak of UV-VIS spectra. In agreement with the result of this study, Krishna et al. [35] reported that MgO nanoparticles shows a specific peak of UV-VIS spectrum at 273.5 nm. In addition, the result indicated that the synthesized MgONFs in this study is new and different from that reported in previous studies [9–21, 30–35] based on its morphology, size, purity, structure, and property, which was evaluated by the integrative analysis of TEM, SEM, XRD, and FTIR.

The result of this study indicated that the synthesized MgONFs at three different concentrations had strong antibacterial activity in both solid and liquid medium against strain GZ 0005 of Xoo, the pathogen of rice bacterial blight. Furthermore, the inhibitory effect of the synthesized MgONFs against Xoo strain GZ 0005 increased with the increase of its concentration. In particular, the inhibitory effect of the synthesized MgONFs was significantly high than that of MgO. Therefore, it can be inferred that the synthesized MgONFs can be used as a promising antibacterial agent against various bacterial plant pathogens, which will protect crops from bacterial diseases.

Nowadays, a lot of hypotheses have been proposed to explain the inhibitory effect of MgO nanoparticles on bacteria. For example, it has been reported that production of reactive oxygen species leads to the release of oxidative stress, which may cause the destruction of cell membranes and the release of ions from the surface of nanoparticles, finally resulting in bacterial death caused by bacterial membrane binding [19]. Furthermore, more and more attention has been paid on the interaction of nanoparticles with bacteria, subsequently damaging the bacterial cell. However, the exact antibacterial mechanism of MgO nanoparticles is still fully unknown.

Our results also indicated that the antibacterial activity of the synthesized MgONFs may be due to its inhibitory effect on bacterial growth, biofilm formation, and swimming motility, which has been found to be highly associated with bacterial survival and virulence. In agreement with the result of this study, Radzig et al. [36] reported that nanoparticles may cause the inhibition of movement and swimming of pathogenic bacteria and also reduce the formation of biofilms. Furthermore, our results indicated that the damage of cell membranes contributed to the antibacterial activity of the synthesized MgONFs against Xoo strain GZ 0005. This result was similar to that of Stoimenov et al. [37], who found that membrane damage may correlate with the production of oxidative stress on cell membranes, eventually leading to inhibition of cell growth and then bacterial death.

## 5. Conclusions

In summary, we successfully synthesized the MgONFs with a size less than 20 nm, which had strong antibacterial activity against rice bacterial blight pathogen Xoo. The MgONFs synthesized in this study was further characterized by analysis of XRD, SEM, TEM, and FTIR. The antibacterial activity of MgONFs against Xoo strain GZ 0005 may be, at least partially, attributed to its inhibition on bacterial growth, biofilm formation, and motility as well as the destruction of cell wall. Finally, it can be concluded that the green synthesis of the MgONFs is a safe method that can be widely used in agricultural applications to suppress rice bacterial disease.

## Data Availability

The data used to support the findings of this study are available from the corresponding author upon request.
